# Interventional Ultrasound in Dermatology: A Pictorial Overview Focusing on Cutaneous Melanoma Patients

**DOI:** 10.1002/jum.16073

**Published:** 2022-08-03

**Authors:** Antonio Corvino, Fabio Catalano, Anna Cipolletta Campanile, Giulio Cocco, Andrea Delli Pizzi, Fabio Corvino, Carlo Varelli, Orlando Catalano

**Affiliations:** ^1^ Motor Science and Wellness Department University of Naples “Parthenope” Naples Italy; ^2^ Radiology Unit Varelli Diagnostic Institute Naples Italy; ^3^ Pathology Unit Varelli Diagnostic Institute Naples Italy; ^4^ Unit of Ultrasound in Internal Medicine, Department of Medicine and Science of Aging University “G. D'Annunzio” Chieti Italy; ^5^ Department of Innovative Technologies in Medicine and Dentistry University “G. d'Annunzio” Chieti Italy; ^6^ Vascular and Interventional Radiology Department Cardarelli Hospital Naples Italy

**Keywords:** cutaneous melanoma, general ultrasound, intervention, nonvascular interventional radiology, oncologic imaging, ultrasound

## Abstract

Cutaneous melanoma incidence is increasing worldwide, representing an aggressive tumor when evolving to the metastatic phase. High‐resolution ultrasound (US) is playing a growing role in the assessment of newly diagnosed melanoma cases, in the locoregional staging prior to the sentinel lymph‐node biopsy procedure, and in the melanoma patient follow‐up. Additionally, US may guide a number of percutaneous procedures in the melanoma patients, encompassing diagnostic and therapeutic modalities. These include fine needle cytology, core biopsy, placement of presurgical guidewires, aspiration of lymphoceles and seromas, and electrochemotherapy.

 

**Abbreviations**
ECT: electrochemotherapy
FNAC: fine‐needle aspiration cytology
PET‐CT: positron emission tomography‐computed tomography
US: ultrasound

Diagnostic imaging plays a major part in the staging and follow‐up of melanoma and in the post‐treatment assessment of metastatic disease.^1^ Being a not invasive and a not expensive too, high‐resolution ultrasound (US) represents the best imaging modality in the evaluation of melanoma patient superficial abnormalities, including primary melanoma tumor (Figure 1), in‐transit metastatic nodule (Figure 2), regional lymph‐node metastasis (Figure 3), and hematogenous cutaneous metastasis.^1^, ^2^, ^3^, ^4^ The role of the US in the melanoma patient is to help assessing disease extent, formulating prognosis, establishing treatment response, and detecting recurrence. Moreover, real‐time US is the most suitable guide for diagnostic and therapeutic procedures in the melanoma patient. This article focuses on the use of US as a guidance tool for percutaneous procedures in patients with cutaneous melanoma.

**Figure 1:**
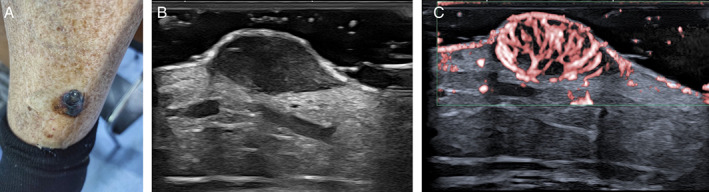
Primary melanoma tumor of the leg. Clinical photograph (**A**). High‐resolution, B‐mode scan showing a homogeneously, hypoechoic thick lesion (**B**). Superb microvascular imaging scan demonstrating an intense tumor vascularization (**C**).

**Figure 2:**
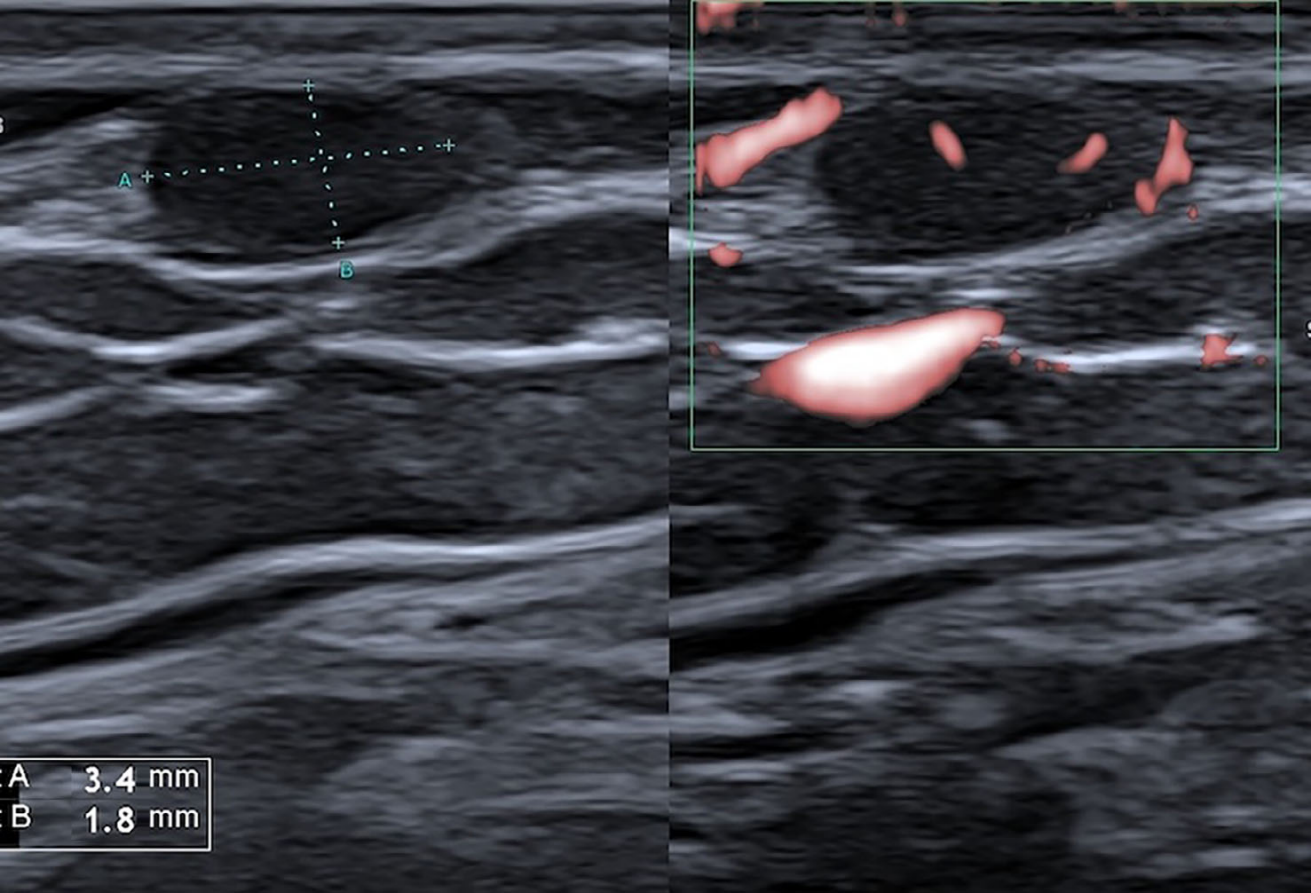
A 3‐mm large, in‐transit melanoma metastasis of the arm. B‐mode (left) and superb microvascular imaging scan (right). Ultrasound allows depicting the small, hypoechoic, subcutaneous nodule and demonstrating the presence of flow signals inside the metastasis.

**Figure 3:**
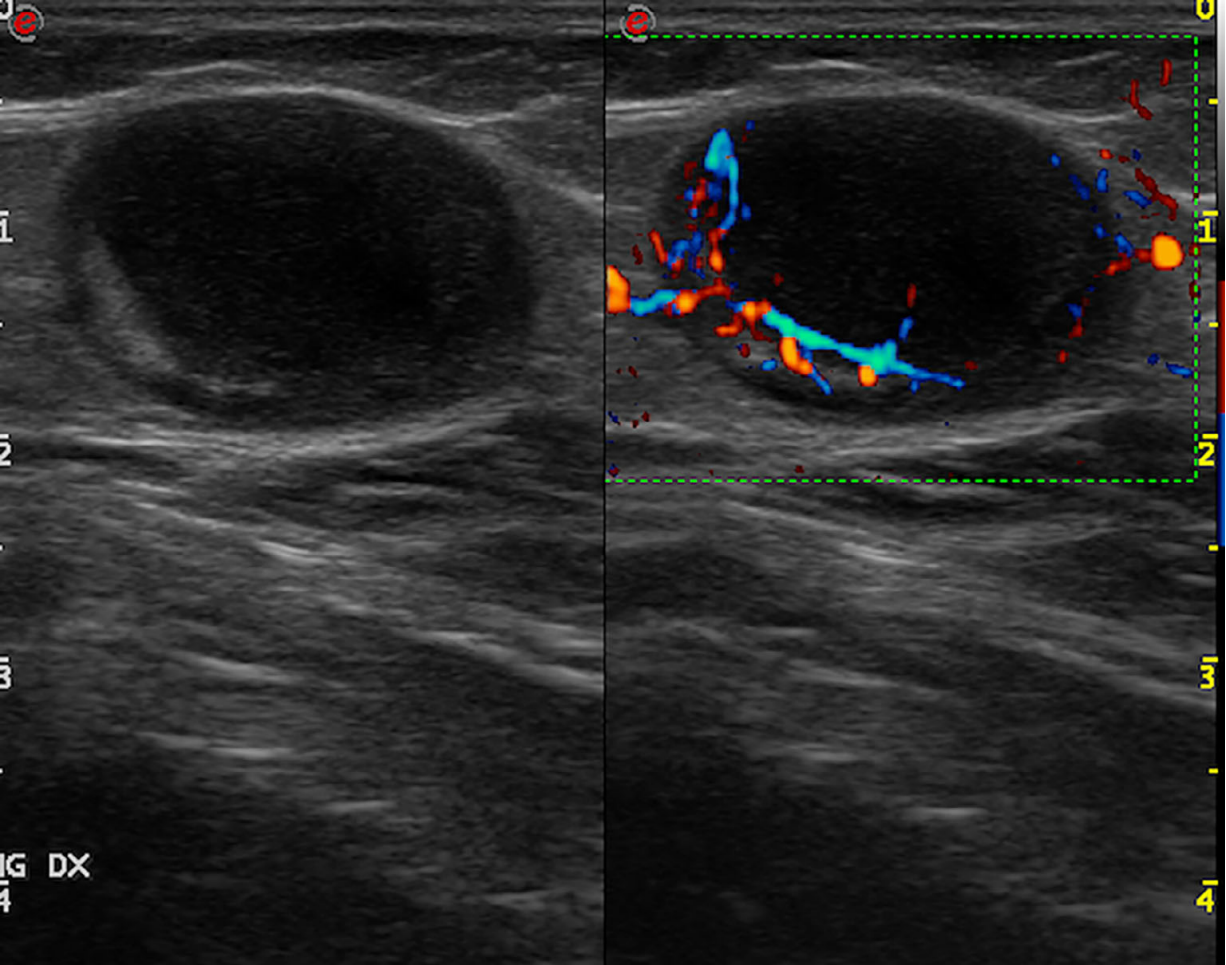
Regional lymph‐node melanoma metastasis to the groin. B‐mode (left) and directional power‐Doppler imaging scan (right). Large, hypoechoic tumor deposit displacing the anatomic and the vascular hilum.

## High‐Resolution US Imaging

Dermatology US requires the use of broadband, high‐frequency transducers. According to the international guidelines, a frequency of at least 15 MHz is mandatory.^5^, ^6^ In clinical practice a frequency range between 20 and 30 MHz works quite well. Higher frequencies offer a remarkable detail of the epidermis and dermis but have a low penetration capability.^3^ The probe must be hold in a firm but gentle way, floating over a large amount of US gel.^3^ Scans are performed on the longitudinal and transverse plane of each abnormality and a special attention is devoted to the perpendicularity of the probe over patient skin, particularly during melanoma lesion measurement.^3^ Power‐ and color‐Doppler scanning is always included, provided that the scanner is set adequately to detect slow flows.^3^ Elastography has not entered the practice of skin US yet, but the preliminary scientific results are quite encouraging.^7^, ^8^, ^9^, ^10^ Qualitative and quantitative assessment with elastography may be of interest for the differential diagnostic assessment of melanocytic skin tumors.^8^ However, measuring the strain ratio has not proven sufficiently accurate in distinguishing thin and thick melanoma.^7^, ^8^

## Fine‐Needle Aspiration Cytology

**Figure 4:**
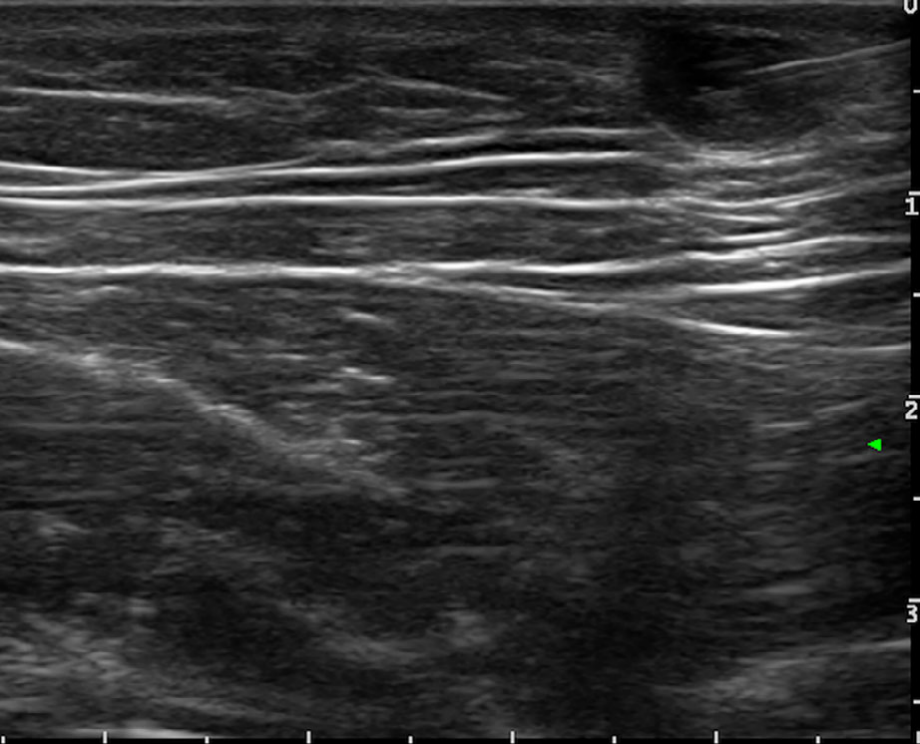
Ultrasound‐guided fine‐needle aspiration cytology of an in‐transit melanoma metastasis.

**Figure 5:**
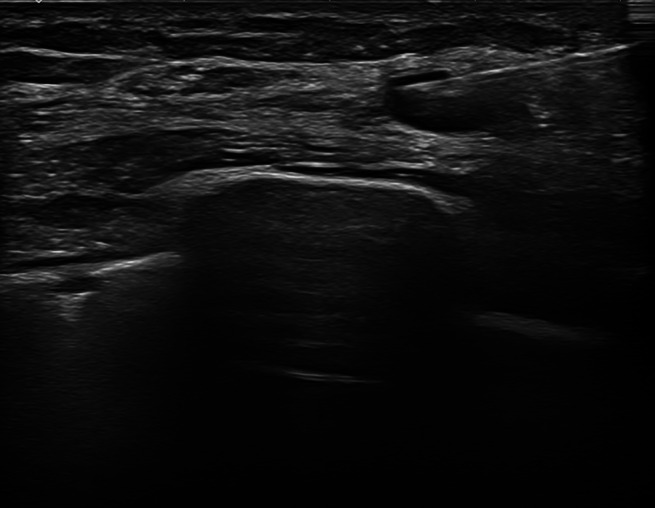
Ultrasound‐guided fine‐needle aspiration cytology of a regional lymph‐node melanoma metastasis to the axilla.

Fine‐needle aspiration cytology (FNAC) can be employed for assess superficial lesions suspected to be melanoma metastases (Figure 4) or US‐abnormal regional lymph nodes, ruling out or confirming melanoma involvement (Figure 5).

Sonographic guidance has proven to be more effective than palpation‐guided FNAC.^10^, ^11^ US with color‐ and power‐Doppler imaging allows to indicate the most suspicious lymph node within a given lymphatic station, to drive the needle toward small, not palpable targets, and to show if the needle tip is really located within the lymph node. Consequently, the number of false‐negative decreases.^4^, ^10^, ^11^ In selected cases, both contrast‐enhanced US and elastography may be helpful in selecting the most suspicion lymph node within a given nodal station or to target the most suspicion area inside a given lymph node.^12^, ^13^ A positive sampling of a suspicious lymph node may also allow avoiding the complex and expensive sentinel lymph‐node excision biopsy and going straight to radical lymphadenectomy.^14^

US employment must be targeted by guiding the needle toward the most suspicious area of the lymph node, such as the hypoechoic focal thickening of the cortex or the most vascularized areas. This allows avoiding intralesional areas at a greater risk of having a necrotic content, such as those with a very hypoechoic texture and without Doppler flow signals.^13^, ^14^

Routinely, the “freehand” technique is employed, by using small diameter needles (21–22 gauge) attached to a 10‐mL plastic syringe for superficial lesions and a 22‐gauge spinal needle for deeply located lesions.^15^ Some authors prefer using thinner needles, between 25 and 27 gauge.^16^, ^17^ Use of larger needles is not associated with a greater risk of biologically active tumor seeding, which represents in any case an anecdotal occurrence after FNAC in patients with melanoma.^18^ Instead, a larger needle allows sampling of a slightly greater amount of material, consequently decreasing the need for sample repetition. During FNAC an active aspiration is carried out when the needle tip is seen within the target. Some authors, however, rely on passive suction (capillarity), unless the target is particularly small.^17^ This theoretically avoids fragmentation of the aspirated material and presence of excessive blood traces. To‐and‐fro, multidirectional movements of the needle inside the target are mandatory for an optimal result.

**Figure 6:**
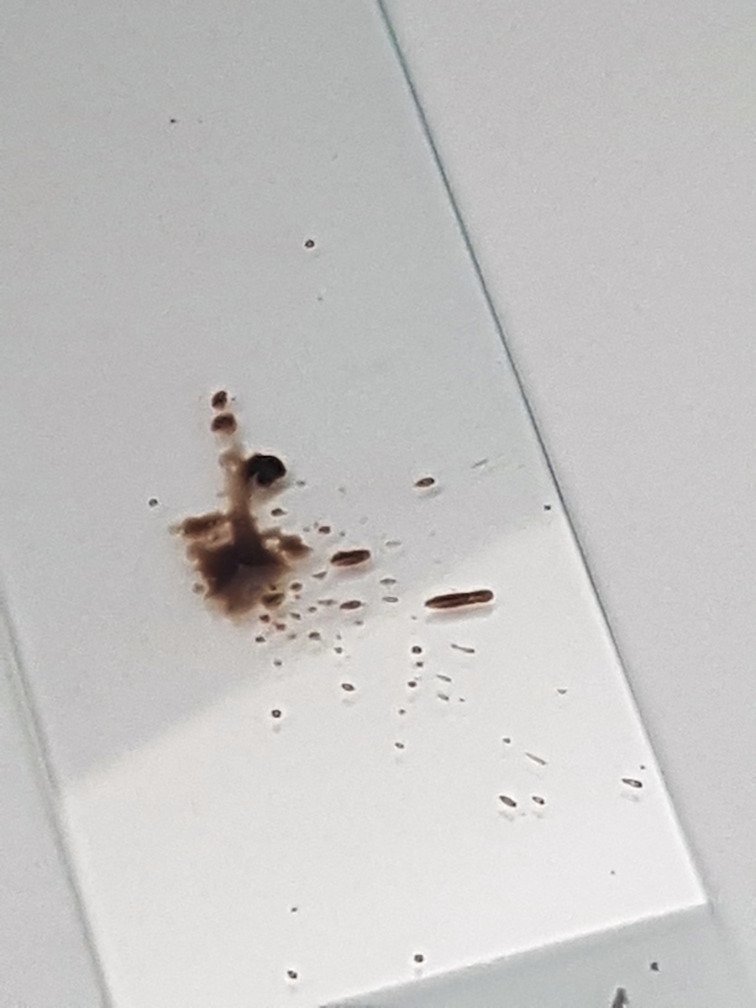
Gross appearance of the fine‐needle aspiration cytology smear of the slide. The brownish appearance is already diagnostic for melanoma metastasis.

**Figure 7:**
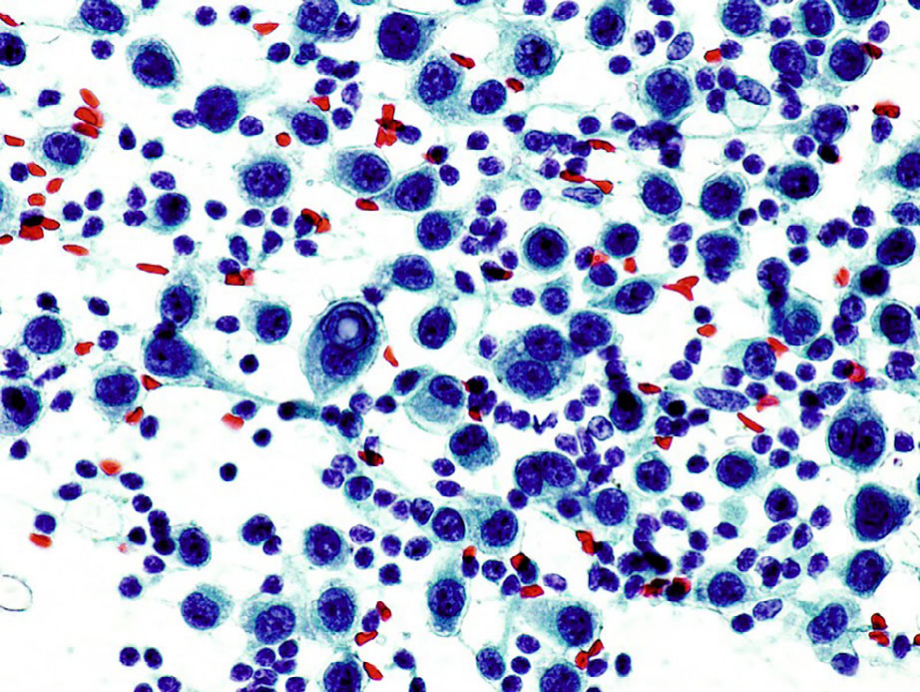
Lymph‐node metastatic, epithelioid melanoma (PAP 60×). Tumor cells show a marked nuclear pleomorphism, prominent nucleoli, and nuclear pseudoinclusions.

**Figure 8:**
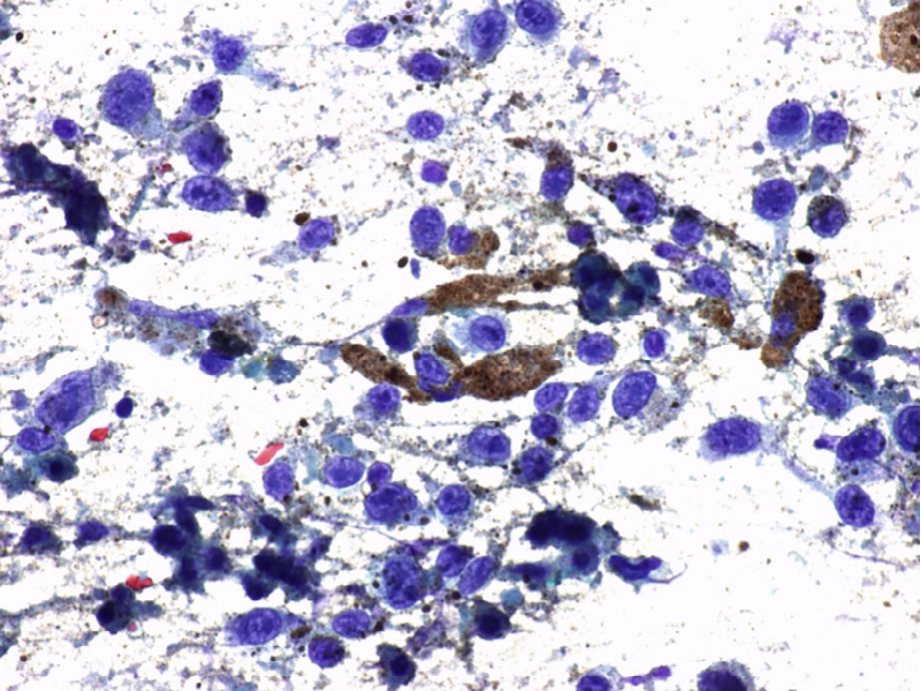
Lymph‐node metastatic, pigmented melanoma (PAP 60×). Neoplastic cells are characterized by marked nuclear pleomorphism, prominent nucleoli, and intracytoplasmatic deposits of melanic pigment.

When the sample material is deployed on the slide, a brownish color is frequently noted in melanoma lesions. Differently from other tumors, such appearance of the aspirated material can be considered already diagnostic (Figure 6). If possible, FNAC samplings are extemporarily evaluated by the on‐site cytopathologist from air‐dried slides with rapid Romanowsky coloration. The puncture is repeated immediately in cases of indeterminate or unrepresentative smears as well as when there is a discrepancy between the clinical, sonographic, and cytologic findings. Definitive cytopathologic assessment from Papanicolaou staining will confirm the preliminary diagnosis (Figures 7 and 8). Immunocytochemical analysis is an extremely useful in the cytopathological assessment, with special reference to the melanocyte marker HMB‐45. Overall, according to literature data, US‐guided FNAC diagnostic performance is very high, with a sensitivity of 98% and a specificity of 100%.^11^

Histologic assessment may be carried out for uncommon cases with indeterminate FNAC findings. Some authors have employed core biopsy with Tru‐Cut needles instead of FNAC to assess suspected lymph nodes in melanoma patients.^19^

## Post‐Surgical Collection

**Figure 9:**
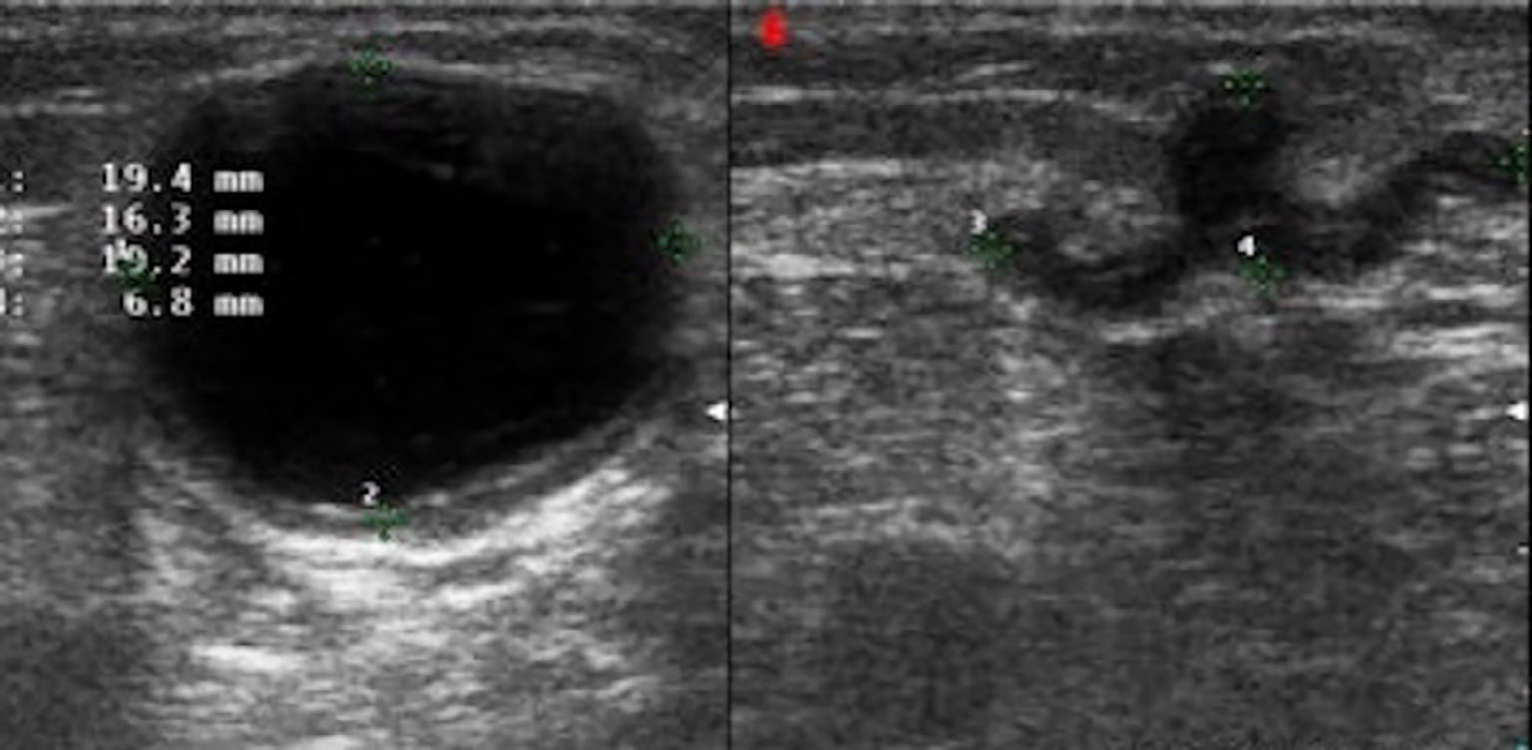
Ultrasound (US)‐guided seroma aspiration. US scan before and after percutaneous aspiration of the 2‐cm collection.

After sentinel lymph‐node excision biopsy or radical lymphadenectomy, melanoma patients may develop fluid collections, including hematomas, seromas, and lymphoceles. In many cases, it is the surgeon himself or herself to perform fluid aspiration on a clinical guidance. However, particularly if the collections are deep or adjacent to critical anatomical structures, an US guide is preferred (Figure 9). A percutaneous aspiration can also have a diagnostic significance, particularly if small fluid collections are identified during patient follow‐up, by detecting any tumor recurrence at an early stage.

## Pre‐Surgical Targeting

**Figure 10:**
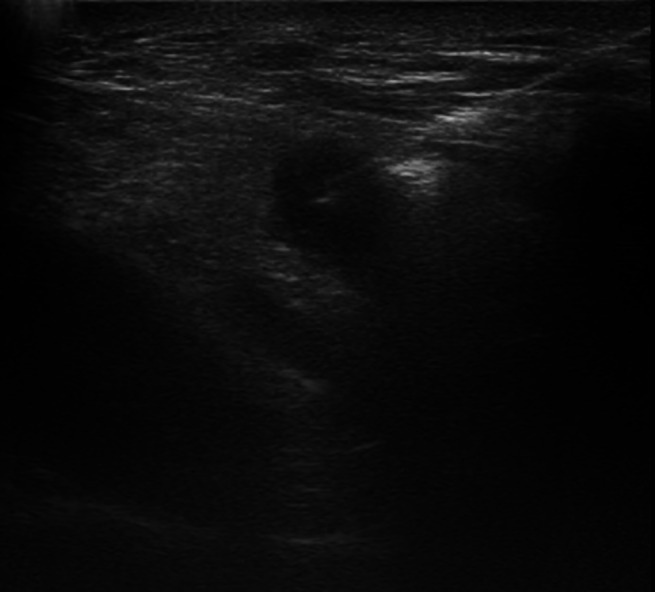
Ultrasound‐guided placement of a pre‐surgical guidewire in a supraclavicular metastasis.

**Figure 11:**
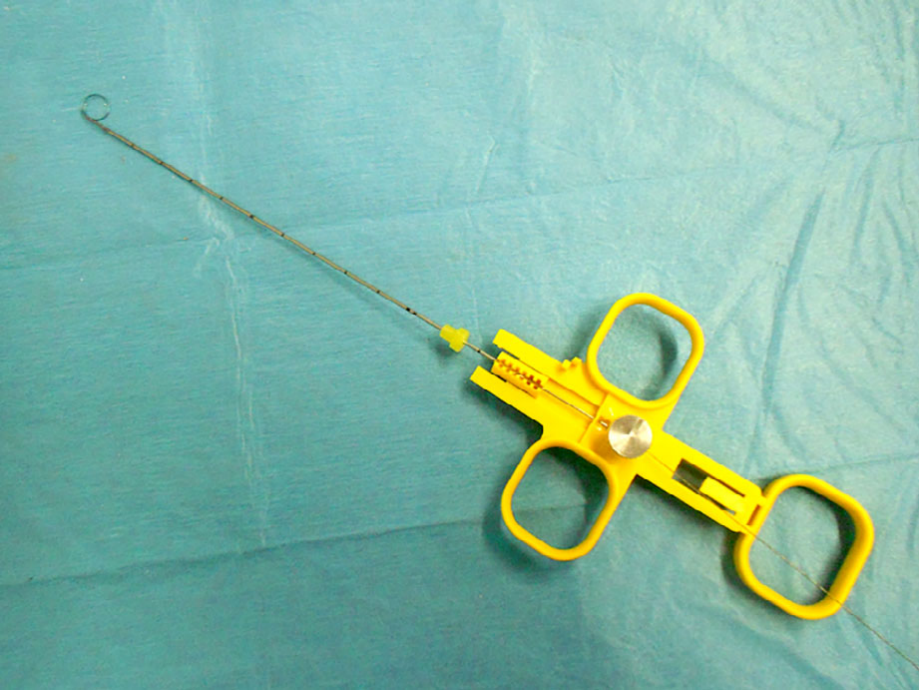
Repositionable guidewire device.

Another extremely useful clinical application of US is the presurgical targeting of melanoma metastases. In cases of deep or small loco‐regional metastasis, skin marking by using a dermographic pencil or a preoperatively percutaneous guidewire placement can be obtained^20^, ^21^ (Figure 10). Pencil marking is reserved to superficial although not well‐palpable lesions while guidewire are employed in case of deeply located abnormalities, particularly if they are located close to vulnerable anatomical structures. A variety of guidewires is commercially available. The most commonly employed sets consist of an outer 18‐gauge needle, of 90‐ or 120‐mm length, and an inner marking wire. Repositionable systems are also available. These wires are more expensive and more complex to manage but, if their placement is not in the perfectly desired point, they can be retrieved and repositioned while non‐repositionable system can only be removed surgically (Figure 11).

Without using any local anesthesia, the tip of the wire is directed under US guidance toward the melanoma lesion. After recognizing the tip in the right position, the needle is minimally drawn back in order to unfold the wire ends, then shaped as an anchor. Finally, the needle is removed and the wire fixing at skin level. Then the patient was referred to the surgeon, who simply had to remove the marked melanoma lesion in local anesthesia.^20^, ^21^

To our knowledge there is no data available to know if placement of a marker wire preoperatively may affect patient survival, causing seeding or predisposing to systemic metastasis. Studies on this aspect would be useful to definitively remove any concern on the use of guidewires in the melanoma patients.

## Therapeutic Procedures

Electrochemotherapy (ECT) is a local, nonthermal treatment combining the use of electric pulses with intravenous or intratumoral injection of anticancer agents to improve drug diffusion into solid cancer cells. Although basically palliative, ECT is a safe and effective treatment for cutaneous and subcutaneous melanoma metastasis.^22^, ^23^ An accurate positioning of electrodes within non‐palpable nodules requires intraoperative US guidance.^23^, ^24^ Solivetti et al^25^ evaluated 15 melanoma patients scheduled to be treated with ECT for in‐transit metastasis. US could detect all the 52 lesions, positron emission tomography‐computed tomography (PET‐CT) 43% of them and telethermography 28%. PET‐CT also yielded a 4% false‐positive rate. Interestingly, Argenziano et al^26^ designed a new type of chitosan‐shelled nanobubbles for the delivery of siRNA‐mediated inhibition of nuclear factor E2‐related factor 2 in combination with an ultrasound. This novel approach can be attractive to overcome chemoresistance in melanoma cells.

## Conclusions

US is playing a growing role in the loco‐regional staging, pre‐sentinel lymph node biopsy nodal screening, and follow‐up of patients with cutaneous melanoma. To make US effective in the melanoma patient, state‐of‐art equipment, specific operators training, careful examination, and interdisciplinary cooperation are needed.^1^, ^27^ Radiologists involved in the melanoma patient management must have expertise in performing US‐ or CT‐guided percutaneous procedures, including fine needle cytology, core biopsy, placement of presurgical guidewires, aspiration of lymphoceles and seromas, and ECT.^28^, ^29^
